# Psychological Interventions for Working with Trauma and Distressing Voices: The Future Is in the Past

**DOI:** 10.3389/fpsyg.2016.02035

**Published:** 2017-01-12

**Authors:** Craig Steel

**Affiliations:** School of Psychology and Clinical Language Sciences, University of ReadingReading, UK

**Keywords:** voices, posttraumatic stress disorder, schizophrenia, dialogue, cognitive therapy

The relationship between stressful or traumatic life events and the content of experiences associated with a diagnosis of schizophrenia is clinically intriguing but lacks developed theoretical understanding. The high prevalence of traumatic events in this group indicates the need to develop psychosocial interventions. However, antipsychotic medication remains the frontline treatment within most mental health services, frequently prescribed by a doctor implicitly (or explicitly) imposing a simplistic disease model, along with the associated lack of hope for those who do not respond well. The essence of this model being that the distressing experiences associated with the diagnosis, typically paranoia and/or hearing voices, are abnormal and symptoms of a disease. Staying within a diagnostic approach, it is worth noting that recent studies suggest that ~15% of people diagnosed with schizophrenia will also present with experiences consistent with a diagnosis of posttraumatic stress disorder (PTSD; Achim et al., 2011). To date the clinical trials aimed at treating PTSD within this group suggest that exposure and eye movement desensitization and reprocessing (EMDR) are effective (Van den Berg et al., 2015) whilst cognitive restructuring alone may not be (Steel et al., 2017). It is likely that psychological interventions for this group will evolve alongside developments in evidence-based interventions for PTSD.

Whilst 85% of people diagnosed with schizophrenia do not fulfill the diagnostic criteria for PTSD, the majority have suffered stressful and traumatic life events (Grubaugh et al., 2011). For many of these people, there appears to be a relationship between their life events and the content of their “psychotic” experiences. For example, Hardy et al. (2005) showed that whilst 12.5% of a sample heard distressing voices which were a direct repetition of a past traumatic event, 45% reported a broader emotional link, e.g., hearing a voice content which made them feel humiliated, replicating the emotional state they experienced during the trauma. There is a need to develop trauma informed approaches for this group, especially when considering the negative relationship between adverse life events and antipsychotic treatment outcome (Hassan and De Luca, 2015).

## The cognitive model and working with voices

The current dominant approach underlying evidence-based psychological interventions is the cognitive model. One strength of this approach is that it is based on some basic premises which can be shared with recipients, thus promoting collaboration. Key premises are that adverse childhood events shape the development of core beliefs about oneself and the world, that thoughts determine emotions, and that behavior maintains beliefs (Beck, 1979). These premises underlie the development of all cognitive behavioral interventions, including cognitive behavioral therapy for psychosis (CBTp). Whilst clinical trials have produced impressive results within CBT for anxiety disorders such as panic and social phobia, the outcomes of CBTp trials have been modest with an effect size typically in the region of 0.4 (Wykes et al., 2008). One area of CBTp promising to move beyond this modest effect is within interventions for the socially disabling experience of paranoia (see Freeman and Garety, 2014). This work sits comfortably within the cognitive model, whereby paranoid beliefs can be formulated as threat beliefs which arise within the context of adverse and traumatic life events.

A recent meta-analysis suggests that the effect size of CBT for distressing voices is small to moderate, and much the same as the outcomes of CBTp for general psychotic symptoms. A key premise in this work is that the interpretation of a voice hearing experience determines the emotional reaction. Making sense of a voice as malevolent, omniscient and powerful is associated with distress. This approach has been evaluated within the recent COMMAND trial (Birchwood et al., 2014), which produced an effect size of 0.57 for the main outcome of “compliance with the voice.” Whilst CBTp for voices is capable of producing significant change, it is not clear what opportunity there is for further significant clinical development within interventions based on the cognitive model.

There are promising developments which attend to the relational aspect of voice hearing (e.g., Hayward et al., 2014). However, it can—and should—be debated whether the underlying premise of these approaches is consistent with the cognitive model. This is not just an academic debate. It is important to clarify what is, and what is not CBTp, to avoid the term becoming so broad and theoretically detached that it has lost all meaning. Defining what constitutes CBTp is particularly important when considering dissemination. It would seem reasonable to suggest that CBTp interventions are ones that are primarily based on the theoretical premises underlying the approach.

Although lacking an established theoretical basis, the relationship between traumatic life events, voice hearing and dissociation is often cited (Pilton et al., 2015). A state of dissociation includes derealization and detachment and can be described as a separation of mental processes which normally work together, but have moved toward independent functioning. Several models of PTSD refer to people entering a dissociative state during a traumatic event. This is argued to be functional and protective at the time of a trauma, but is also the source of information processing changes which underlie the development of trauma-related intrusions, or “flashbacks.” High levels of dissociation have been observed within individuals diagnosed with schizophrenia (Ross and Keyes, 2004), with some clinical researchers proposing a “dissociative trauma” subtype of schizophrenia and associated treatment implications. It has also been argued that dissociation mediates the relationship between traumatic life events and hearing voices (Varese et al., 2012). However, in part due to the lack of theoretical development of the concept of dissociation itself, we are some way short of a theory which is of any clinical utility when working with the content of distressing voices. However, a voice can be conceptualized as representing a dissociated “part of the self” (Corstens et al., 2012), which would suggest that engagement with the content of the voice may be of therapeutic value.

## Talking with the “enemy”

As noted above, the contents of voice-hearing experiences are rarely repetitive memories of a traumatic event. They often seem to be linked to past experiences within a context that does not sit easily within the cognitive model. Clinicians often refer to a voice being linked to a life event, but that the content and communication has “evolved” beyond the specific event which is considered to be the trigger. The premise that voices “arrive” in peoples' lives as part of a meaningful reaction to traumatic life events, and that voice content is relevant and should be engaged with (including active “Voice Dialogue”), underlies an approach put forward by Romme and Escher (2000) often called the “Maastricht approach.” The approach has become established within the hearing voices movement but has largely remained outside academic investigation and mainstream clinical services. Another important premise within the Maastricht approach is that a voice is serving a function. Therefore, active engagement with the voice may reduce conflict between “parts of the self.” This form of voice dialogue work invites the voice to communicate directly with the therapist, often with the voice-hearer sitting in a different chair when communicating as “the voice.” The therapist is respectful to the voice, whatever the voice may be believed to represent, and can maintain a dialogue over a number of sessions with the aim of exploring the voice's function. Once the function is understood the clinical work can move toward the wider issues within the voice hearers' life, with reduced direct communication with the voice. For example, during dialogue, a voice may state that it shouts and is aggressive because it has become tired of being ignored. A voice may reveal that it became “active” during a period of abuse in someone's life, and that at the time it was trying to help. However, the voice goes on to say that the reason it currently sounds critical of the voice-hearer is because it wants them to stand up for themself, and to “kill” the part of them that behaves like a victim.

As noted in the literature, it is not uncommon for a voice hearer to hear the voice in the form of a past abuser or torturer. In this circumstance, therapists are often anxious that dialogue with the voice may retraumatize the voice hearer. However, through voice dialogue, the voice hearer is likely to realize that the voice represents a view about the abuse and is not actually the abuser talking to them. It is worth drawing a parallel with psychological therapy for depression. The negative internal thoughts associated with depression are (usually) considered to have developed through adverse life events and a direct confrontational approach to the content of these thoughts is typically considered to be cognitive therapy done badly. If a voice is part of an individual, and the content also linked to past events, then it would seem that direct confrontation is also ill-advised in this context. Further, it can be argued that to avoid voice dialogue work is to collude with the voice hearer, that the voice is powerful and to be feared.

As argued above, it is important to acknowledge the premises upon which interventions are based. There are clear differences between the underlying premises of CBT for voices and the Maastricht approach. A misplaced absorption of the latter within the former is likely to impede the development of the Maastricht approach. Further, the two approaches would advocate fundamentally different positions when working with some voice hearers. It is not uncommon for a voice hearer to report a voice which undermines and criticizes the voice hearer. Although there is no single approach to this scenario within CBT for voices, common strategies include distracting oneself from the experience, and tackling the negative voice content by talking back to it with assertive statements. These strategies would seem diametrically opposite to opening a meaningful dialogue, in order to understand the function behind the voice and to reduce conflict. Within the Maastricht approach, the process adopted within some forms of CBT for voices would be considered likely to increase internal conflict and the associated distress. There is, therefore, an opportunity to empirically test these predictions. To date, voice dialogue and the Maastricht approach have been reported in the context of single cases (Corstens et al., 2012) and an ongoing case series (ISRCTN54370851).

Research aimed at the development of the Maastricht approach would best start with a focused assessment of voice dialogue. As within clinical trials of medication, there is the need for the close monitoring of adverse events when evaluating psychological interventions. Pre–post dialogue assessment and feedback from voice hearers are also required. However, ahead of larger scale clinical trials, there is the need to develop two important aspects of wider research into voice hearing. One is the need for measures that better reflect the priorities of voice hearers. Second, is the need for criteria to define meaningful subgroups of voice hearers so as to inform appropriate intervention. The Maastricht approach is most likely to be appropriate for the frequently mentioned “trauma dissociative” subgroup which requires further distinction.

In summary, there is increased awareness that voice hearing is linked to life events, but a lack of theoretical development has restricted clinical development. The role of appraisal which is embedded within the cognitive model has proved to be clinically useful, but we seem to have reached the limit of what can be offered from this approach. We should resist the temptation to categorize an increasing range of interventions under a broad definition of CBT. Distinct interventions should be recognized as such and evaluated independently. Although voice dialogue does not sit within an established theoretical framework, it does offer a distinct approach to working with voice content which warrants systematic evaluation. It is worth noting the current widely held perception of voice hearing as part of normal human experience was held back by the mainstream mental health professions during the twentieth century. It is hoped that the same assumptions do not inhibit the widespread acceptability of voice dialogue.

## Author contributions

The author confirms being the sole contributor of this work and approved it for publication.

### Conflict of interest statement

The author declares that the research was conducted in the absence of any commercial or financial relationships that could be construed as a potential conflict of interest.
